# Cervical spine fractures in osteopetrosis: a case report and review of the literature

**DOI:** 10.7555/JBR.32.20170055

**Published:** 2018-01-26

**Authors:** Arjang Ahmadpour, Amir Goodarzi, Darrin J. Lee, Ripul R. Panchal, Kee D. Kim

**Affiliations:** Department of Neurological Surgery, University of California-Davis Medical Center, Sacramento, CA 95817, USA.; Department of Neurological Surgery, University of California-Davis Medical Center, Sacramento, CA 95817, USA.; Department of Neurological Surgery, University of California-Davis Medical Center, Sacramento, CA 95817, USA.; Department of Neurological Surgery, University of California-Davis Medical Center, Sacramento, CA 95817, USA.; Department of Neurological Surgery, University of California-Davis Medical Center, Sacramento, CA 95817, USA.

**Keywords:** osteopetrosis, spine, fractures, osteoclasts, operative management

## Abstract

While management of appendicular fractures has been well described in the setting of osteopetrosis, there is limited information on managing fractures of the axial spine. Here we present an osteopetrotic patient with multiple traumatic multiple, comminuted, unstable cervical spinal fractures managed with non-operative stabilization, and provide a review of the pathophysiology, genetic characteristics, and special considerations that must be explored when determining operative *versus* non-operative management of spinal injury in osteopetrosis. A PubMed query was performed for English articles in the literature published up to June 2016, and used the following search terms alone and in combination: "osteopetrosis", "spine", "fractures", "osteoclasts", and "operative management". Within four months after initial injury, treatment with halo vest allowed for adequate healing. The patient was asymptomatic with cervical spine dynamic radiographs confirming stability at four months. On four-year follow up examination, the patient remained without neck pain, and CT scan demonstrated partially sclerotic fracture lines with appropriate anatomical alignment. In conclusion, external halo stabilization may be an effective option for treatment of multiple unstable acute traumatic cervical spine fractures in patients with osteopetrosis. Given the challenge of surgical stabilization in osteopetrosis, further research is necessary to elucidate the optimal form of treatment in this select patient population.

## Introduction

Osteopetrosis, variably referred to as marble bone disease and Albers-Schönberg disease, was first described in 1905 by the German radiologist, Heinrich Albers-Schönberg. Schonberg described osteopetrosis as a group of inherited skeletal abnormalities characterized by severe osteoclastic dysfunction^[1– 6]^.

Osteoclasts are multinucleated cells derived from stem cells within the bone marrow. They play a crucial role in the resorption and remodeling of bone in the normal skeleton^[7]^. Defects in osteoclastic function can result in the formation of fragile bone, despite an increase in total bone mass. Thus, as a result of poor bone quality, patients with osteopetrosis frequently suffer from skeletal fractures, bony deformity, osteosclerosis, osteomyelitis, compressive neuropathies, hematopoietic dysfunction, and stunted growth^[8– 9]^.

A common presentation of patients with osteopetrosis is spinal fractures following minor trauma. Treatment of such cases remains controversial, without any concrete evidence-based therapeutic guidelines. Stable fractures without major neurologic compromise have been treated conservatively with external orthosis, whereas unstable fractures have been treated with either external orthosis or surgical fixation^[10]^. Although there are published cases in which external orthosis was successfully used to manage fractures in osteopetrotic patients, there are no documented cases of halo orthosis used for treatment of acute traumatic cervical fractures. We present a case of multiple, comminuted, unstable cervical spinal fractures in osteopetrosis treated successfully with halo orthosis, and a review of the literature on this challenging pathology.

## Case report

A 55-year-old female with a history of osteopetrosis, multiple orthopedic fractures, chronic osteomyelitis of the jaw, congenital monocular blindness, and chronic anemia presented to the emergency department after a high-speed motor vehicle collision. Upon arrival, the patient complained of neck pain and right chest wall pain without any motor or sensory deficits. On neurological exam, she had full motor strength in her upper and lower extremities. She denied any acute changes in bowel or bladder function.

**Fig.1:**
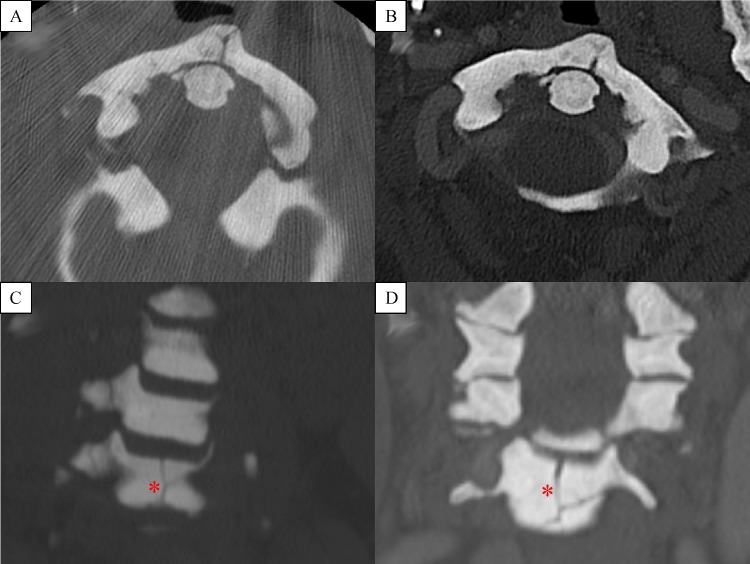
Axial CT cervical spine sequences demonstrate healing of the C1 anterior arch fracture at the time of injury (A: 2011) in comparison to four-year follow up images (B: 2015).

**Fig.2:**
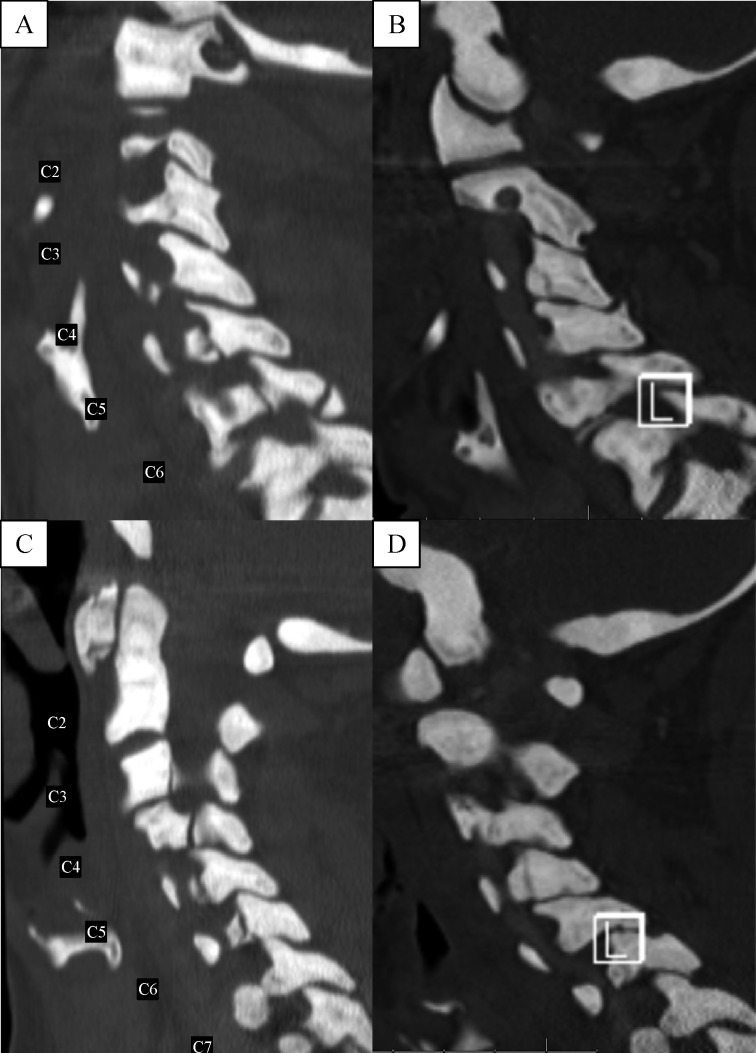
CT cervical spine sagittal sequences.

The initial computed tomography (CT) scan of the cervical spine revealed numerous fractures including a C1 anterior arch fracture, bilateral C3 pedicle fractures extending through the transverse foramina, bilateral C4 pedicle fractures extending through the transverse foramina and right lamina, C5 vertebral body fracture oriented through the anterior/inferior aspect of the vertebra with bilateral pedicle fractures extending to the transverse foramina and right lamina, and bilateral C6 pedicle fractures extending through the transverse foramina and right lamina (***Fig. 1*** and ***2***). Given the extensive involvement of the anterior, middle and posterior columns, the fractures were deemed to be unstable. Given the history of osteopetrosis and presumed poor bone quality, the decision was made to utilize external cervical halo orthosis.

**Fig.3:**
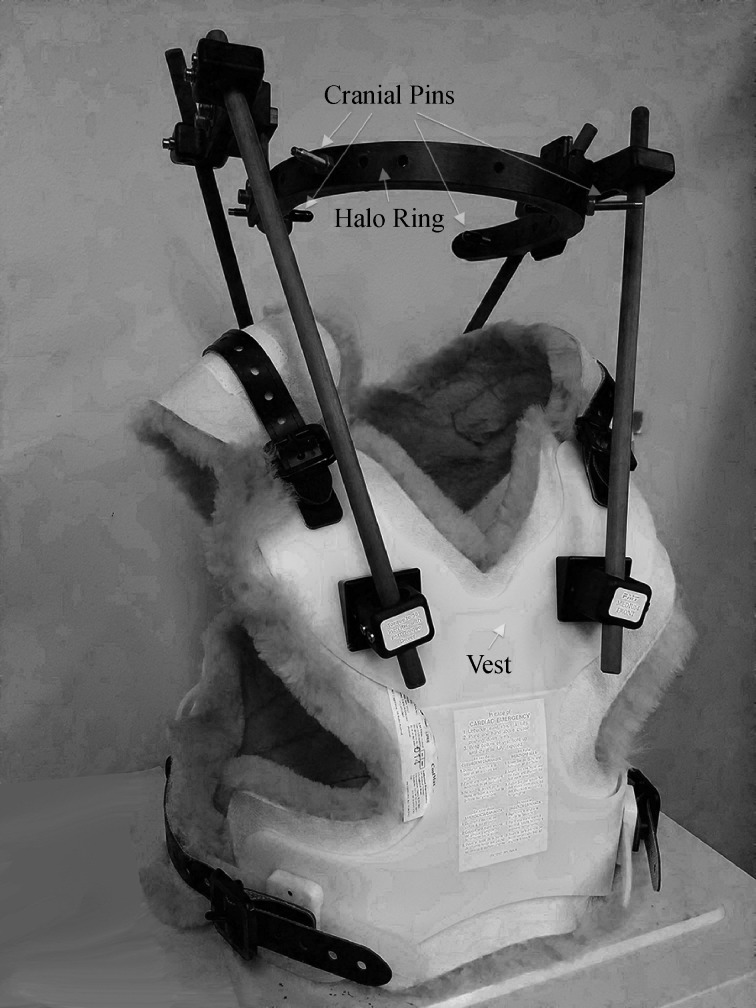
An assembled Halo Ring and Vest that was utilized for our patient.

**Fig.4:**
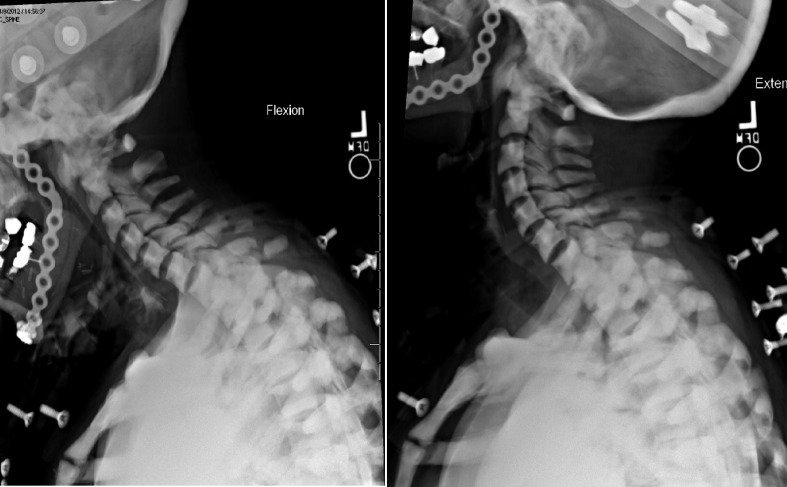
Cervical spine flexion and extension radiographs.

The patient was evaluated four months after initial injury without any neck pain or neurologic deficits. Flexion extension films were obtained after removing the halo vest, demonstrating minimal grade 1 spondylolisthesis at C6–C7. The halo ring (***Fig. 3***) was subsequently removed (***Fig. 4***).

On follow up examination, four years after the initial injury, the patient remained asymptomatic without any neck pain. Repeat CT scan at this time demonstrated sclerotic old fracture margins with normal anatomical alignment (***Fig. 1***).

Furthermore, a PubMed query was performed for English articles in the literature published up to June 2016, and used the following search terms alone and in combination: "osteopetrosis", "spine", "fractures", "osteoclasts", and "operative management". Initially, 3,176 articles were found. Review articles, and prospective and retrospective studies were identified, in which osteopetrosis was discussed. The references from all of the selected articles were then further examined to identify additional suitable studies. Overall, 273 articles met our initial inclusion criteria before we applied the following exclusion criteria: (1) Data that was redundant with other later reviews of the literature 2) Case reports with no review of the literature; were not included in the discussion. There were 69 articles remained after applying these exclusion criteria.

## Discussion

### Physiology

In healthy bone, osteoblasts synthesize bone matrix, and osteoclasts resorb bone. Osteoclast attachment to bone is made possible by podosomes with filamentous actin and α_v_β_3_ integrin. These molecules associate with the matrix proteins osteopontin and vitronectin on the surface of bone^[11– 13]^. To achieve acidification of the resorption lacunae and to initiate bone demineralization, carbonic anhydraseⅡproduces bicarbonate and protons from carbon dioxide and water^[11, 14]^. These protons are then transported across the membrane *via* active transport through the osteoclast-specific vacuolar-type H^+^-ATPase proton pump. This process generates a pH of approximately 4 to 5 in the extracellular space adjacent to targeted bone^[11, 15]^. This acidification in the extracellular environment initiates the breakdown of bone, mainly hydroxyapatite^[11]^.

### Pathophysiology

Ordinary bone growth is regulated by a balanced formation of bone by osteoblasts, and bone resorption by osteoclasts^[11]^. In osteopetrosis, the resorptive process of osteoclasts is disturbed, with a resultant imbalance in bone remodeling^[11, 17– 22]^. This failure of adequate osteoclasts resorption results in excess bone growth, and contributes to many structural and functional defects of the skeletal anatomy. The body experiences a decreased ability to create an acidic environment in extracellular compartment, thus decreasing the resorption of bone and calcified cartilage. The overall result is a generalized sclerosis (abnormal hardening of bone)^[17, 23]^.

The histopathology of osteopetrosis demonstrates woven bone architecture, widened trabeculae, and diaphysis calcification^[17, 24– 25]^. These histologic features of osteopetrotic bone result from deficient remodeling during nascent development of bone^[17]^. The dysfunctional osteoclasts fail at resorbing woven bone, primary spongiosa, and the calcified cartilage in immature bone^[17, 24– 25]^. Failure of primary spongiosa absorption during development gives rise to the sclerotic appearance of osteopetrotic bone on radiograph. The thick trabeculae and calcified cartilage that remain in the diaphysis of bone has mechanical properties that are inferior to that of healthy bone, and lead to pathological fractures^[17]^.

### Genetics

The term osteopetrosis encompasses a heterogeneous group of heritable conditions^[11]^. This rare disorder's overall incidence is unknown. The more severe manifestation of this disease is inherited either in an autosomal recessive (incidence of 1:250,000) or X-linked manner and is usually fatal during infancy and early childhood^[1– 2]^. Conversely, patients with the autosomal dominant (incidence of 5:100,000) form of osteopetrosis have a normal life expectancy with a more benign clinical course. These patients are mostly susceptible to orthopedic complications including: axial skeleton fractures, long bone fractures, bony deformity, osteosclerosis, osteomyelitis, and compressive neuropathies^[3]^.

The molecular origin of autosomal recessive osteopetrosis (ARO) in humans has been reviewed in detail^[26– 32]^. Seven different genes play a role in the pathogenesis of ARO^[27]^. Four of the seven genes (TCIRG1, CLCN7, PLEKHM1, OSTM1) undergo loss of function mutations that lead to osteoclast-rich osteopetrosis^[27, 33– 37]^. These osteoclasts do not possess morphological defects, however, they do not form the ruffled border needed for the resorption of bone^[49]^. Two of the seven genes (TNFSF11, TNFRSF11A) undergo loss of function mutations resulting in osteoclast poor forms of ARO^[27, 38– 40]^. Together, TCIRG1 and CLCN7 mutations are responsible for approximately 70% of ARO cases^[27, 41– 46]^.

The exceedingly rare intermediate form of autosomal recessive osteopetrosis (IRO), results from a loss of function of the *CAII* gene^[14]^. This results in a defect of the carbonic anhydrase II protein expressed in erythrocytes^[47– 48]^. IRO is associated with renal tubular acidosis and has highly variable clinical signs in patients^[14, 49]^.

Autosomal dominant osteopetrosis (ADO) exhibits a milder disease course and is seen with greater frequency in adults^[22, 49]^. ADO patients may exhibit delayed healing, increased fracture occurrence, and osteomyelitis^[1, 17, 20– 22]^. ADO was formerly sub-classified as type I and type II. Recent investigations demonstrate that the disorder previously termed type I dominant osteopetrosis is attributed to defects in the LRP5 gene with a resultant increased osteoblast bone deposition. This is not associated with a defect in osteoclasts, but rather, should be considered LRP5-activating bone disease or high bone mass phenotype^[11, 22, 50]^. For this reason, ADO now refers to what was previously described by Albers-Schönberg, and discovered to be a form of Chloride Channel 7 deficiency osteopetrosis stemming from a single- allele dominant negative mutation in the CLCN7 gene^[11, 21– 22, 49]^. However, it is important to note that a CLCN7 mutation has not been confirmed in up to 30% of patients with an ADO clinical phenotype^[22, 49, 51]^.

Currently, there are no established genotype-phenotype correlations for ADO^[22, 43, 52]^ and there is no established explanation for the reduced penetrance that ranges from 66 to 94%^[20– 22, 53– 55]^.

Osteopetrosis can also present with an X-linked recessive inheritance pattern caused by a mutation in the IKBKG gene on the X chromosome^[30, 41]^. X-linked recessive osteopetrosis is exceedingly rare, and more frequently seen in males. In addition to the pathologically dense bones seen in the ARO and ADO forms of the disease, X-linked osteopetrosis can attribute to lymphedema, anhidrotic ectodermal dysplasia (which effects hair, skin, sweat glands, and teeth), and immunodeficiency^[30, 42]^. OL-EDA-ID is an acronym derived from each of the major features of X-linked osteopetrosis, and is often used to refer to X-linked osteopetrosis.

### Radiographic characteristics

Radiographic findings of osteopetrosis include marked thickening of the cranial vault, sclerosis of the skull base, vertebral endplate thickening leading to "Rugger Jersey" appearance of the spine, and sclerosis of axial skeleton and the pelvis^[3]^. Many ADO patients' radiographs exhibit sclerotic foci located within ossification centers, referred to as endobones^[9, 56]^.

There is some degree of radiographic spinal involvement in nearly all reported cases, but some patients exhibit uniformly dense or uninvolved vertebral bodies^[2, 9, 57– 60]^. Despite the common presence of radiographic endplate thickening of the vertebrae in ADO, and that thoracic or lumbar scoliosis develops in 25% of cases, few reports of spinal deformity exist in the literature^[9, 20, 56, 61]^.

### Operative management

Reports of operative treatment are scarce and no gold standard procedures are recognized in the treatment of osteopetrotic fractures^[62]^. When surgical stabilization is required, poor bone quality should be anticipated with unpredictable post-operative course in terms of arthrodesis and overall clinical outcome^[15]^. Osteopetrotic bone quality increases the risk of marrow cavity disruption and iatrogenic fractures. Prevention of these potential pitfalls requires drilling at a low speed with spaced cycles and cold saline irrigation, frequent drill bit changes, and avoiding mallet usage^[63– 65]^. Close follow-up at 1 week, and 1, 3, 6, 9 and 12 months has been recommended to monitor the higher risks of infection and re-injury^[63]^.

Surgical instrumented stabilization has been reported with moderate success in unstable fractures. Auerbach *et al*. reported a case of acute traumatic C2 TypeⅡ odontoid fracture in a 21-year-old woman with osteopetrosis^[16]^. Evidence of non-union was exhibited by flexion extension films that showed instability at C1-2^[16]^. The patient was managed using C1-2 transarticular screw arthrodesis. It was reported that due to the fragile nature of the bone continuous fluoroscopy was necessary to meticulously advance the screws as to avoid iatrogenic fractures. Autograft was harvested from the patient's ileum, which was complicated with an iatrogenic fracture at the harvest site. Interspinous wiring and morcellized allograft bone and demineralized bone matrix were utilized to maximize early stabilization and rate of arthrodesis. Post-operatively, the patient was kept in a hard cervical collar for three months and within 18 months had complete resolution of her neck pain (***Table 1***)^[5]^. This case highlights the technical difficulty and intraoperative risk of iatrogenic fractures in this patient population.

**Tab.1:** Reported cases of patients with osteopetrosis and acute traumatic cervical spine fractures

Author	Age (year)	Gender	Fracture location	Outcome
***External Fixation***					
Armstrong et al. (1999)	4	Female	C2	Pedicle	Complete resolution of fracture and neck pain
Armstrong et al. (1999)	9	Female	C2	Posterior Arch	Complete resolution of fracture and neck pain
***Surgical Fixation***					
Aurbach et al. (2007)	21	Female	C2	Odontoid (TypeⅡ)	Complete resolution of fracture and neck pain

Martin *et al*. reported a seven-year-old boy with spondylolysis at the C2 and C3 vertebra and pain in both regions^[66]^. The patient exhibited cervical spine instability on flexion and extension radiograph, and underwent a C1-C4 modified Gallie's posterior cervical arthrodesis with autogenous bone graft from the iliac crest. A Philadelphia collar was used for 12 months postoperatively because the authors felt the child would not tolerate other collar types^[66]^. This patient did not undergo fusion, but experienced resolution of pain at the six-month follow up, and was asymptomatic at the 37 month follow up. Armstrong *et al*. reported a case of a 29-year-old man with severe spondylolithesis and pain refractory to conservative management^[15]^. The patient was treated with reduction and posterior fusion with Harrington rods and a fibular strut graft^[15]^.

### Non-operative management

The occurrences of cervical and lumbar pathology related to osteopetrosis have been previously reported, and most of these cases were managed non-operatively^[5, 9– 10, 14– 15, 66]^. Armstrong *et al*. reviewed the management of acute cervical spine fractures in two children with osteopetrosis using external stabilization^[15]^. The first patient was a four-year-old girl with a traumatic non-displaced C2 pedicle fracture and the second patient was a nine-year-old girl with a traumatic C2 posterior arch fracture. Both patients were managed with external cervical orthosis with complete healing of fractures and resolution neck pain (***Table 1***)^[15]^.

Martin *et al*. described five patients who had cervical and lumbar spondylosis^[10, 66]^. Three of these patients with isolated lumbar spondylosis were treated with an orthosis or corset^[66]^. Szappanos *et al*. reported the occurrence of lumbar spondylolisthesis and spondylolysis in five patients, of which two presented with back pain^[5]^. These two patients were effectively treated with lumbar corsets^[5]^. Suzuki *et al*. reported a case of a woman with bilateral L4 vertebral arch fractures^[10, 67– 69]^. She was treated with immobilization for three months in a body cast with successful healing of the fracture^[10, 67– 69]^.

After careful consideration of our patient's poor bone quality, risk of non-union, numerous fractures, and high likelihood of surgical complications, we proposed the use of external cervical stabilization. It was felt that the use of a cervical collar would not provide adequate stabilization given the involvement of all three columns and the multitude of fractures, thus we opted for a halo orthosis. The patient tolerated the procedure and had no complications related to the pin sites or the halo vest. Flexion and extension films were obtained four months after halo placement (***Fig. 4***) and showed minimal spondylisthesis at C6-7 without signs of gross instability. At that time the patient's neck pain had completely resolved and the halo was removed. The patient was seen for a follow up visit 4 years after initial injury, and remained pain free. ***Fig. 1 ***demonstrates side by side four-year follow up axial CT images of the C1 anterior arch fracture with a partially healed fracture line with normal anatomical alignment and C5 vertebral body fracture showing persistent fracture lines with sclerotic bony margins. This is an example of poor bone remodeling and healing found in patients with osteopetrosis. However, ***Fig. 2 ***reveals at four-year follow up sagittal CT images of minimally displaced C3, C4, C5, C6 pedicles and C6 spinous process fracture showing adequate healing fracture lines and normal anatomical alignment.

Although further investigation is necessary to find the optimal treatment of acute traumatic cervical spine fractures in osteopetrosis, this case demonstrates that halo fixation may be a viable option for patients with multiple acute traumatic cervical fractures.

## Conclusion

External halo stabilization may be an effective option for treatment of unstable acute traumatic cervical spine fractures in patients with osteopetrosis. Given the challenge of surgical stabilization in osteopetrosis, further research is necessary to elucidate the optimal form of treatment in this rare patient population.
